# Natural Epithelial Barrier Integrity Enhancers—*Citrus medica* and *Origanum dayi* Extracts

**DOI:** 10.3390/gels10120836

**Published:** 2024-12-19

**Authors:** Sarah Coopersmith, Valeria Rahamim, Eliyahu Drori, Rachel Miloslavsky, Rima Kozlov, Jonathan Gorelick, Aharon Azagury

**Affiliations:** 1 The Department of Chemical Engineering and Biotechnology, Ariel University, Ariel 4070000, Israel; 2 Eastern Research & Development Center, Kiryat Arba, Ariel 40700, Israel

**Keywords:** chemical penetration enhancers, nanoparticles, biomimetics, buccal drug delivery, *Citrus medica*, *Origanum dayi*, hyaluronic acid

## Abstract

Buccal drug delivery offers a promising alternative for avoiding gastrointestinal degradation and first-pass metabolism. However, enhancing the buccal epithelial barrier’s permeability remains challenging. This study explores the effects of ethanolic extracts from *Citrus medica var. Balady* (CM), *Citrus medica var. Calabria* (CMC), and *Origanum dayi* (ORD) on buccal epithelium permeability in vitro using a TR146 cell-based model. The cell viability assay revealed that the extracts were non-toxic at the concentration range tested (<0.5% *w*/*v*). Surprisingly, none of the tested extracts significantly enhanced the buccal permeability of 40 kDa Fluorescein Isothiocyanate Dextran (FD40). However, the CMC and ORD extracts significantly reduced the epithelial permeability of FD40, mirroring the effects of hyaluronic acid (HA), a known barrier integrity enhancer. The total phenolic content (TPC) analysis suggested a potential link between the phenolic concentration and epithelial barrier reinforcement. The rapid colorimetric response method was applied to assess the interaction of these extracts with biological membranes. The results indicated that HA interacts with cellular membranes via lipid bilayer penetration, whereas the extracts likely influence the barrier integrity through alternative mechanisms, such as ligand–receptor interactions or extracellular matrix modulation. These findings highlight the potential of CMC and ORD extracts as natural agents to enhance buccal epithelial integrity. In conclusion, incorporating these extracts into formulations, such as hydrogels, could offer a cost-effective and biocompatible alternative to HA for improving buccal cavity health.

## 1. Introduction

The buccal cavity, constituting the initial segment of the digestive system, encompasses the inner cheeks, palate, and sublingual tissues. Utilizing the buccal route for drug administration offers an advantageous alternative to oral intake, primarily due to its rapid absorption and avoidance of first-pass metabolism. However, the residence time in the buccal cavity is short, which requires fast and efficient drug permeation. Moreover, enhancing buccal mass transport is also necessary due to the low permeability of the buccal cavity. One prominent method for achieving this goal is using penetration enhancers (PEs) [1,2].

A PE refers to a pharmacologically inert chemical agent capable of augmenting the mass transport of drugs across biological tissues or into cells, thereby facilitating their penetration and uptake. The efficacy and safety of PEs vary based on factors such as their concentration, origin (natural vs. synthetic), and intended application, which are pivotal in developing effective and safe PEs [3,4,5]. In recent years, there has been a shift towards natural PEs due to their inherent attributes such as non-toxicity, non-immunogenicity, biodegradability, and biocompatibility. Moreover, natural PEs are readily available, cost-effective, non-addictive, and generally devoid of adverse side effects [6].

As opposed to PEs, several materials have been tested for their ability to increase the integrity of the buccal epithelium (essentially decreasing buccal mass transport). Hyaluronic acid (HA) is one of the most extensively researched materials. HA stimulated tight-junction-related genes including ZO-2, marvelD3, cingulin, claudin-1, claudin-3, and claudin-4 [7,8]. These studies indicate that HA entrapped in hydrogels significantly affects epithelial tight junctions and enhances buccal barrier integrity (as opposed to PEs’ effect on permeability). Additionally, the mucoadhesive properties of HA vary depending on its molecular weight, with lower-molecular-weight HA exhibiting a superior mucoadhesive performance [9,10]. Another example can be found in flavonoids (a type of polyphenolic material) such as alpinetin, icariin, and quercetin, which have also been shown to enhance epithelial barrier function by upregulating tight junction proteins, modulating signaling pathways involved in barrier regulation, and reducing inflammation, thereby increasing epithelial barrier integrity [11].

*Citrus medica*, one of the oldest fruits mentioned in the Bible, is rich in polyphenols and exhibits potent antioxidative and antibacterial effects [12]. Moreover, it contains various flavonoids known for their beneficial properties. In vivo and in vitro research has highlighted the significant contribution of *Citrus* flavonoids to preventing degenerative and infectious diseases [13]. Another genus known for its therapeutic activity is *Origanum*, with some species known to contain high concentrations of monoterpenes [14], which can disrupt bacterial cell membranes, leading to alterations in membrane permeability and the leakage of intracellular materials. For example, *Origanum dayi* has demonstrated an antiproliferative effect on various cancer cells cultivated in vitro, suggesting its potential in combating cancer cell proliferation [15,16]

The initial purpose of this study was to evaluate the potential of ethanolic extracts of *Citrus medica var. Balady* (CM), *Citrus medica var. Calabria* (CMC), and Origanum Dayi (ORD) as permeation enhancers (PEs) for mass transport across the buccal epithelium. To that end, we first assessed the potential toxicity of these ethanolic extracts on buccal epithelial cells (i.e., the TR146 cell line). Following this, we measured the impact of these ethanolic extracts on the mass transport across layers of TR146 cells—a cell line derived from the buccal mucosa of a 67-year-old female patient [17]. TR146 cells are the standard in vitro model for studying the buccal epithelium [18]. When some of these extracts were found to reduce the mass transport across the buccal epithelium, we compared their effect on buccal mass transport to that of HA—a known amplifier of buccal barrier integrity [8]. Lastly, in an attempt to elucidate the mechanism of action, we analyzed the interaction of the tested ethanolic extracts (and that of HA) with cellular membranes using a rapid colorimetric response [19,20,21]

## 2. Results and Discussion

**Figure 1:**
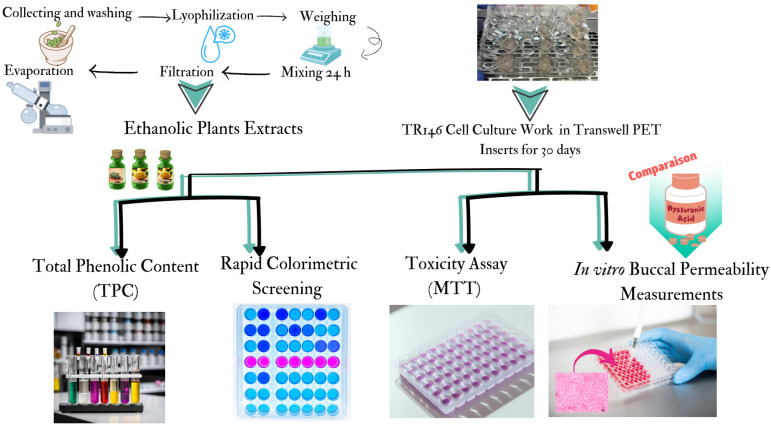
A schematic diagram of the methodology used in this study. Additional details of the applied methods are depicted in the graphical abstract. Ethanolic extracts were tested for their TPC and used in the 3-(4,5-Dimethylthiazol-2-Yl)-2,5-Diphenyltetrazolium-Bromide (MTT) assay, both with and without cells. Extracts’ effect was compared to hyaluronic acid solutions as epithelial barrier integrity preservers in permeability tests and rapid colorimetric screening.

In order to enhance and clarify what was tested and done in this paper, a schematic diagram of the methodology used in this study is presented in Figure 1 below.

### 2.1. The Toxicity Test

**Figure 2:**
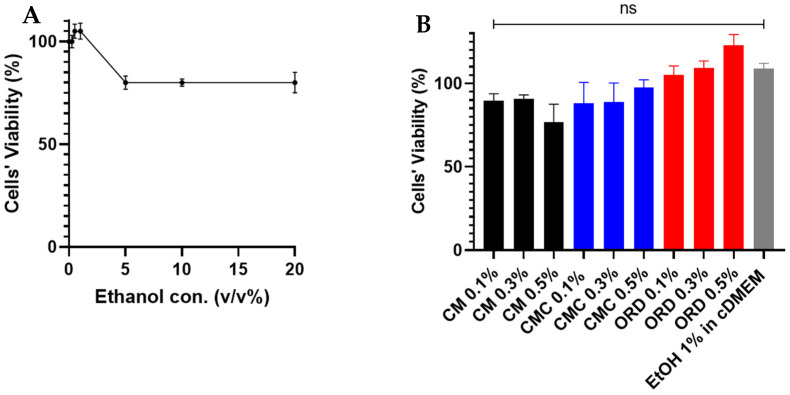
TR146 cell viability post-one-hour exposure to (**A**) increasing ethanol concentrations (0–20% *v*/*v*) in complete medium (cDMEM) and to (**B**) increasing concentrations (0.1%, 0.3%, and 0.5% *w*/*v* in 1% ethanol in cDMEM) of extracts of CM, CMC, and ORD. Values present the means ± SDs of *N* > 2; statistical significance was tested via applying the Kruskal–Wallis test, ns—nonsignificant.

Before assessing these extracts in the form of hydrogels, the potential toxicity of these extracts and ethanol to TR146 cells must be evaluated. To this end, we assessed the viability of TR146 cells via the MTT method after one hour of exposure. The results are presented in Figure 2.

As seen in Figure 2A, the viability of the TR146 cells was not affected by ethanol up to 1% *v*/*v* in cMDEM compared to the control. At 5% to 20% ethanol, the viability of the TR146 cells decreased to about 90%. To be cautious, since ethanol is known as a PE even at small concentrations (1%) in cDMEM (Figure 2A and Figure S4) [5,22], we continued with extracts containing 1% ethanol. In Figure 2B, we can see that the viability of the TR146 cells was not significantly affected by exposure to the extracts—of CM, CMC, or ORD—up to 0.5% *w*/*v* compared to the control (i.e., 1% ethanol in cDMEM). Thus, we continued with these extract concentrations for the following experiments.

### 2.2. In Vitro Buccal Permeability Results

**Figure 3:**
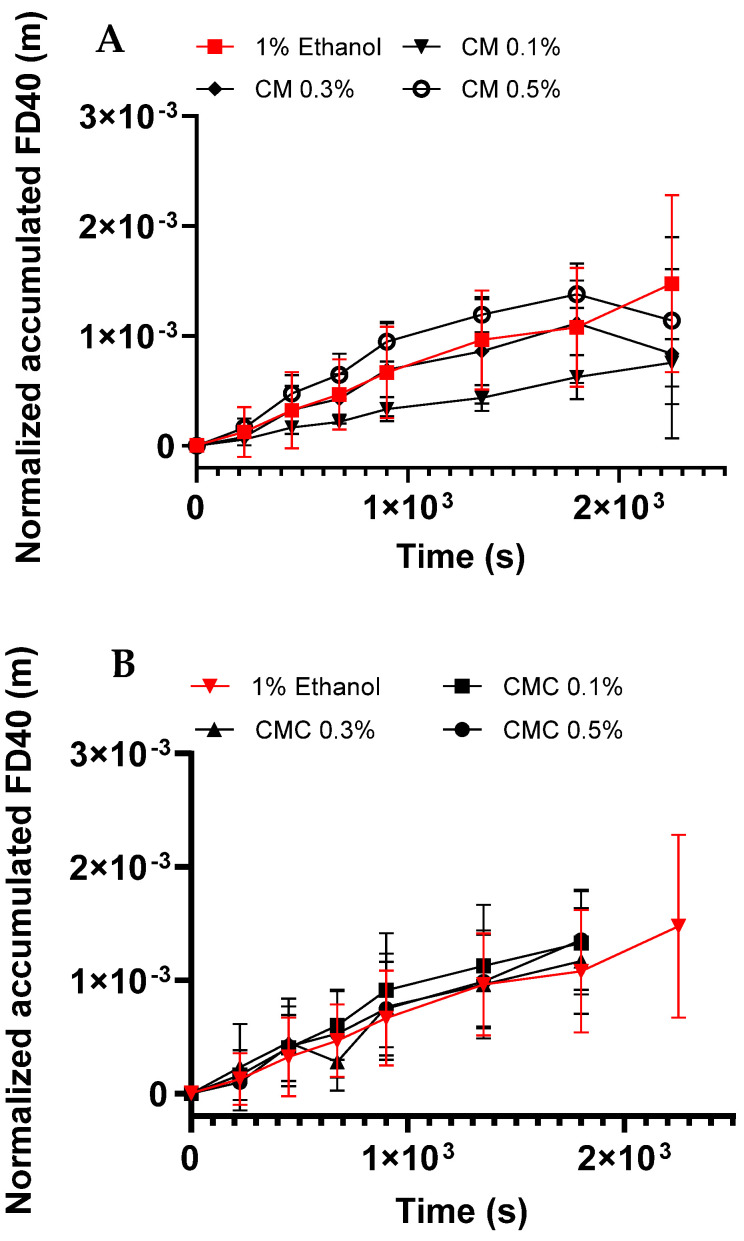

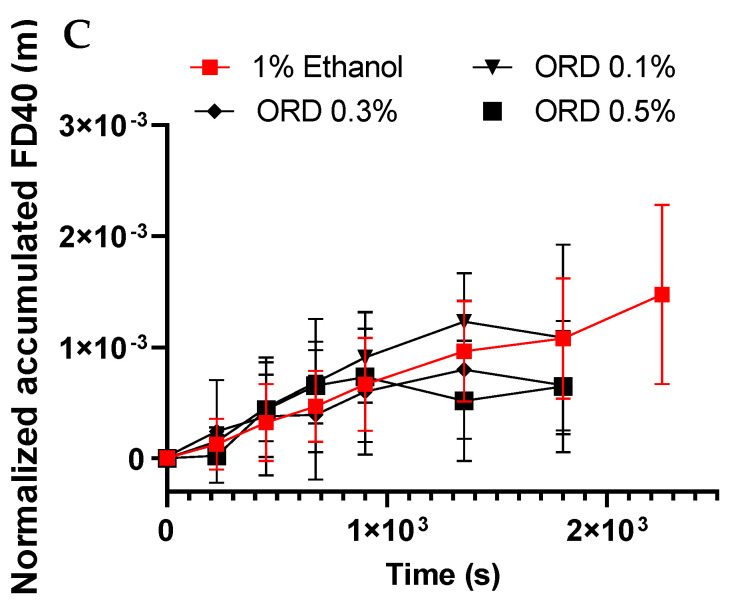
Normalized (see Equation (2)) accumulated FD40 in the receiving chamber that permeated through TR146 layers after one hour of exposure to the tested extracts and control solution (ethanol 1% *v*/*v*, red line) in cDMEM. 0.1%, 0.3%, and 0.5% (*w*/*v*) of (**A**) CM in 1% ethanol in cDMEM, (**B**) CMC in 1% ethanol cDMEM, and (**C**) ORD in 1% ethanol and cDMEM. Values present the means ± SDs of *N* > 3.

Next, layers of TR146 were grown on inserts and used as an in vitro model of the buccal epithelium to assess the effect of the CM, ORD, and CMC extracts on their permeability. At first, the extracts were evaluated as PEs (see Equation (2)) by summating the accumulative FD40 at the bottom receiver compartment after the cells were exposed to the solution tested; the sum was divided by the cross-section of the transwell insert and the initial FD40 concentration in the upper section. Figure 3 shows the normalized accumulated amount of FD40 after one hour of exposure to the tested extract solutions.

In Figure 3A, linearity was maintained for 2 h, indicating the inherent high permeability of the TR146 layers. The sharpest slope was observed for 0.5% CM in cDMEM, followed by 1% ethanol solution, 0.3% CM, and 0.1% CM. For the CMC and ORD extracts (Figure 3B and Figure 3C, respectively), linearity was maintained (for 2 h for CMC extracts and 1.5 h for ORD extracts). Notably, an increase in the concentration of these extracts did not affect the FD40 accumulation in the receiving chamber.

**Figure 4:**
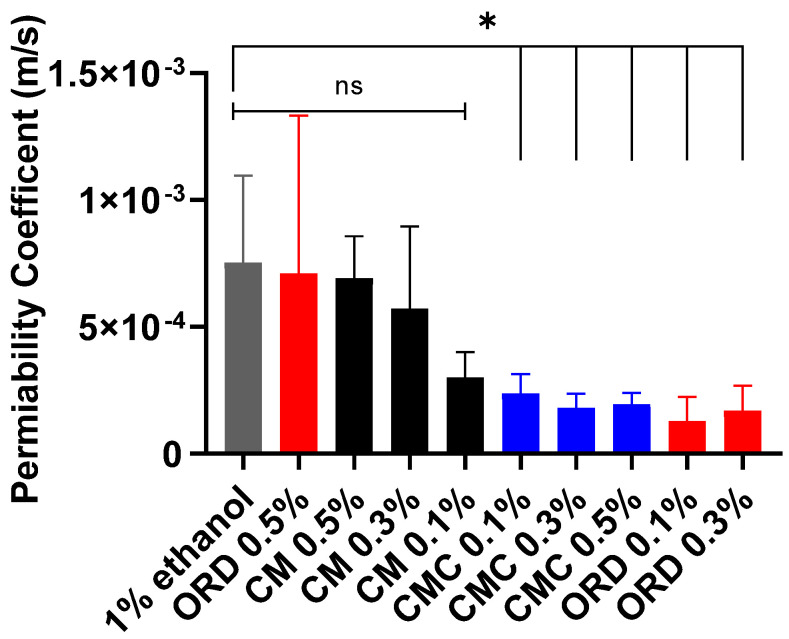
PCs of FD40 cross an in vitro human buccal epithelium (TR146 layers) after exposure to different solutions for 1 h, calculated from the linear region. The statistical analysis involved comparing the calculated PC of 1% ethanol in CDMEM to the PCs of the CM, CMC, and ORD extracts. Values present the means ± SDs of *N* > 3; statistical significance was denoted as * *p* < 0.05 after applying the Brown–Forsythe one-way ANOVA test, ns—nonsignificant.

Next, the respective PC of FD40 across the TR146 layers was calculated from the slopes (seen in Figure 3 and used to compare the effects of the tested solutions on the TR146 layer’s permeability). PC was calculated from the slope of each extract according to Equation (2) and is shown in Figure 4. Statistical analysis was performed by comparing the PCs obtained for the different extracts to the PC of the control solution of 1% ethanol in cDMEM.

As seen in Figure 4, as the concentration of the ORD and CM extracts increased from 0.1% to 0.5%, there was an increase in buccal epithelial permeability (from 1.3 × 10^−4^ m/s to 7 × 10^−4^ m/s for ORD and from 3 × 10^−4^ m/s to 7 × 10^−4^ m/s for CM). On the contrary, for the CMC extract, increasing its concentration from 0.1% to 0.5% did not affect buccal epithelial permeability (still constant at 2 × 10^−4^ m/s). The PCs of TR146 post-exposure to CM (at all concentrations) and ORD (o.5%) for one hour were comparable to the PC of the control solution of 1% ethanol in cDMEM, and as such, they cannot be considered buccal PEs. Interestingly, exposure of the TR146 layers to ORD (0.1% and 0.3%) and CMC (0.1%, 0.3%, and 0.5%) resulted in a statistically significantly lower PC, indicating that these extracts decreased the permeability of the TR146 layers, thus effectively enhancing the integrity of the buccal epithelial barrier.

**Figure 5:**
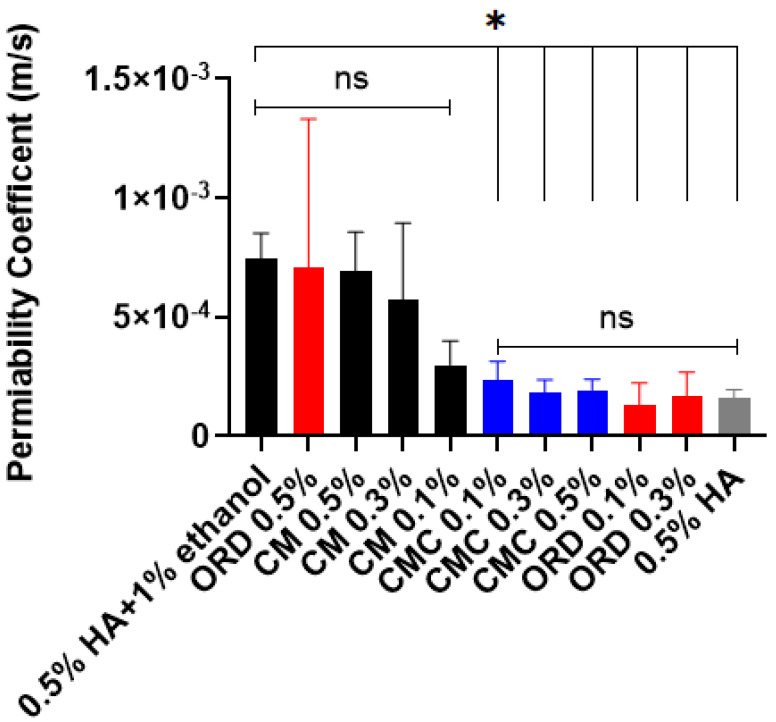
Calculated PCs of FD40 across the in vitro human buccal epithelium (i.e., TR146 layers) after 1 h of exposure to the tested solutions extracted compared to 0.5% (*w*/*v*) HA with and without 1% ethanol in cDMEM. Values present the means ± SDs of *N* > 3; statistical significance was denoted as * *p* < 0.05 after applying the Brown–Forsythe one-way ANOVA test.

Due to this finding, we decided to assess the effect of HA (with and without 1% ethanol in cDMEM) since it is a known enhancer of buccal epithelial barrier integrity [6,9,10,23,24]. This was carried out to enable us to compare the efficiency of HA to that of the tested ethanolic extracts. The results of this comparison are presented in Figure 5.

Figure 5 compares the calculated PCs of FD40 across the TR146 layers of the tested solutions to the PC after exposure to 0.5% HA with 1% ethanol and without ethanol (colored bars) in cDMEM. It can be observed that the PCs obtained post-exposure to CMC (all concentrations) and ORD (0.1% and 0.3%) were found to be four times (0.02 cm/h) lower than the PC post-exposure to 0.5% HA with 1% ethanol in cDMEM (0.074 cm/h). However, none of the extracts tested displayed a statistically significant difference compared to HA applied without ethanol. Thus, we can conclude that CMC (0.1%, 0.3%, and 0.5%) and ORD (0.1% and 0.3%) can be considered epithelial barrier integrity enhancers similar to HA (750–1000 kDa) [19].

### 2.3. Principal Component Analysis (PCA)

PCA was conducted to find an optional correlation between the parameters of the extract concentrations, TPC, and normalized PC. The PCA referred only to the extracts because the solutions of ethanol, hyaluronic acid, hyaluronic acid with ethanol, and cDMEM had constant concentrations and served as control solutions. Therefore, no concentration dependence should be observed.

**Figure 6:**
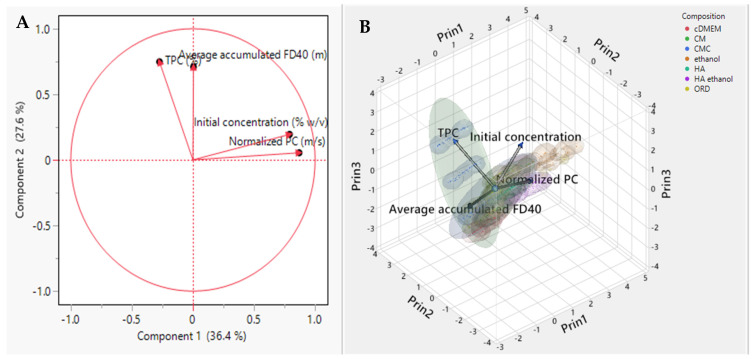
PCA illustrates the contributions of various variables to the principal components derived from PCA for the extracts. (**A**) is in two dimensions considering only two principal components (more than 60% variance), and (**B**) is in three dimensions of three major components (more than 80% variance).

As seen in Figure 6, each vector represents a variable (extract concentrations, TPC, and normalized PC). At the same time, the direction indicates the relationship with the components. The length of a vector (distance from the origin) reflects the strength of that variable’s contribution to the variance explained by the principal components. It can be deduced that all parameters present a strong relationship with components 1 and 2, except for initial concentration, which represents a less strong correlation with component 2.

The average accumulated FD40 shows a strong relationship with both components, exhibiting a positive correlation with TPC and PC and a negative correlation with initial extract concentration. Specifically, the TPC value also rises as the average accumulated FD40 increases. The TPC value is influenced solely by the type of extract, and the PCA indicates that the accumulated FD40 is more dependent on the extract type than on the initial extract concentration.

As discussed in the toxicity test (Section 2.1), extract concentration significantly impacts cell viability, affecting the density of the cell layers growing on the transwell inserts. This density can influence the amount of FD40 accumulated in the bottom receiver compartment, potentially explaining the strong positive correlation between these two variables. Conversely, both “normalized PC” and “initial concentration” exhibit strong correlations with the components but in opposite directions, indicating a weak or negative correlation between these two variables. This suggests that changes in the initial concentration may not proportionately affect the normalized PC. The following question arises: why is PC not strongly correlated with accumulated FD40? The answer lies in the fact that PC depends on the rate of penetration over time. As time increases, PC rises even as a negative correlation develops between time and accumulated FD40.

Next, we wanted to better understand our extracts’ mechanism of action by examining how they interacted with biomimetic membranes via the rapid colorimetric screening method [23].

### 2.4. Rapid Colorimetric Screening Analysis

Rapid colorimetric screening [19] characterized the interaction between the cell membrane’s phospholipids and the extracts tested [19]. The solutions were evaluated based on their initial %CR (percent reduction) at the zero time point and their final %CR after 20 min of incubation with the membrane-mimicking PDA vesicles.

**Figure 7:**
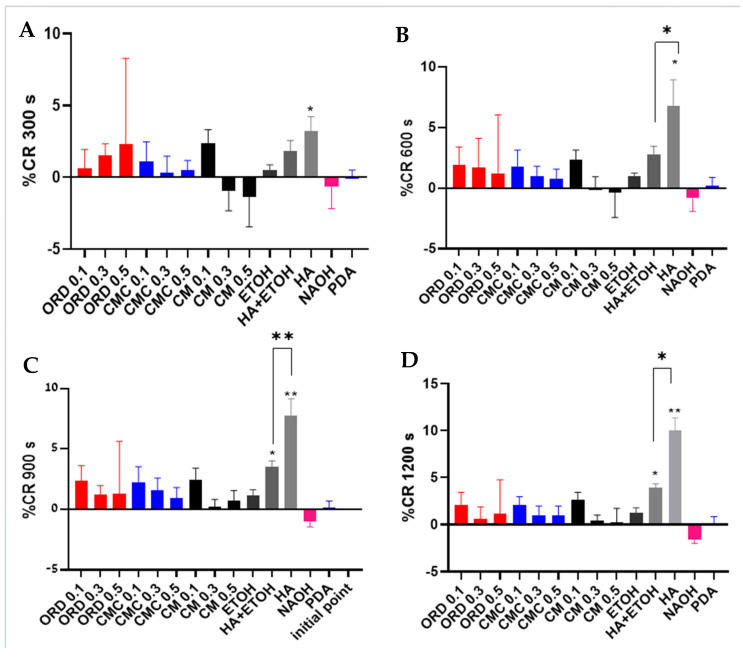
%CR of PDA vesicles during 1200 s of exposure to the tested extracts of ORD, CMC, and CM at concentrations of 0.1%, 0.3%, and 0.5% (*w*/*v*) with 1% (*v*/*v*) ethanol and 0.5% HA with or without 1% ethanol (all in cDMEM). The %CR was measured after (**A**) 300 s, (**B**) 600 s, (**C**) 900 s, and (**D)** 1200 s of incubation with the PDA vesicles. Values represent the means ± SDs of *N* > 4; statistical significance is denoted as * *p* < 0.05 and ** *p* < 0.01.

At the initial point (time 0), only the control solution (NaOH 1M) induced a change from blue to red due to the ionic interactions between Na^+^ and OH^−^ and the PDA chain [19]. However, after 300 s (Figure 7A), 0.5% HA induced a significant color shift from blue to red, while none of the tested extracts demonstrated a considerable %CR. After 900 s, HA with ethanol started to present a considerable %CR (Figure 7C). Significance between the two HA solutions appeared at 600 s (Figure 7B) and was maintained until the endpoint (Figure 7D at 1200 s). This %CR indicates that the highly polar HA penetrates the lipid bilayer of cellular membranes. Note that the %CR of HA in the presence of ethanol was reduced significantly (from 10% to 4%), further supporting the conclusion that ethanol inhibits the interaction of HA with lipids, thus negating its effect on the integrity of the buccal epithelium. When ethanol was also in the solution, the CR% was lower than without ethanol (Figure S8).

Depending on its molecular weight, HA can stimulate tight-junction-related genes, including ZO-2, marvelD3, cingulin, claudin-1, claudin-3, and claudin-4 [11,13]. On the other hand, none of the extracts displayed a significant %CR, which leads to the conclusion that none penetrated the cells’ membranes nor affected the phospholipids’ structure. Therefore, we postulate that effective extracts may affect buccal epithelium integrity by activating cellular cascades. These cascades are activated via interaction with unique ligands on buccal epithelium membranes but not in PDA vesicles. Another option is that the extracts may affect the buccal epithelium by interacting with the extracellular matrix [25].

Alternatively, a paracellular pathway refers to the transport route between adjacent cells, specifically through the tight junctions (TJs) that connect the epithelial and endothelial cells. The permeability of the paracellular pathway can vary and is influenced by various factors, including the composition of tight junction proteins such as claudins and occludin, as well as external factors like inflammatory cytokines and dietary components such as polyunsaturated fatty acids (PUFAs) [23]. Observing the results of the colorimetric reactions, we can assume that the extracts tested herein can improve the tight junctions between TR146 cell membranes since they do not interact with the phospholipid bilayer of the cells but enhance the buccal integrity (as was presented in Figure 5). In summary, the paracellular pathway is a critical aspect of epithelial barrier function, allowing for selective transport while maintaining the integrity of the epithelial layer [23].

## 3. Conclusions

This study aimed to examine the effects of ethanolic extracts of two types of *Citrus medica*, as well as *Origanum dayi*, on the mass transport of FD40 across the buccal epithelium. First, we found that these ethanolic extracts did not affect the viability of TR146 cells, suggesting they are not toxic and could be safe to use. Next, these extracts were assessed as PEs of the permeability of TR146 layers in vitro, where, surprisingly, none of the tested extracts showed a significant effect compared to the control solution of 1% ethanol in cDMEM.

However (and quite interestingly), CMC (0.5%, 0.3%, and 0.1%) and ORD (0.1% and 0.3%) worked contrarily to our expectations and decreased the mass transport across the buccal epithelium. Thus, we compared the effect of these specific extracts on the PCs of FD40 to the impact of high-MW HA (750–1000 kDa)—a known and proven buccal epithelial barrier integrity enhancer—as a reference. We found that the PCs of these extracts were ~4-fold (5.5 × 10^−8^ m/s) smaller than the PC obtained post-exposure to 0.5% HA with 1% ethanol in cDMEM (5.5 × 10^−8^ m/s vs. 2.05 × 10^−7^ m/s, respectively). Moreover, through rapid colorimetric screening, we deduced that HA interacts with lipid membranes by penetrating the phospholipid bilayer. At the same time, the extracts may affect the buccal epithelium by activating cellular cascades through attachment to specific ligands or interaction with the extracellular matrix or by modulating the TJs through the paracellular pathway.

We also wanted to evaluate whether there was any relation between the TPC in the tested extract and buccal integrity; after running a PCA, we found that the average accumulated FD40 exhibited a positive correlation with TPC. This finding indicates that the phenol concentration in the extract may affect buccal integrity.

To conclude, this study’s results showed that CMC and ORD extracts may improve and strengthen the integrity of the buccal epithelium. Moreover, these extracts could serve as effective and cheaper alternatives to HA for enhancing buccal cavity health.

## 4. Materials and Methods

### 4.1. Materials

Epithelial TR146 cells were obtained from the European Collection of Authenticated Cell Cultures (ECACC); passages 8–12 were used for this study. Dulbecco’s Modified Eagle’s Medium (DMEM) with high glucose (4.5 g/L) and without phenol red, Fetal Bovine Serum (FBS), Penicillin–Streptomycin (PS) antibiotic, L-glutamine solution, Dulbecco’s Phosphate-Buffered Saline (PBS), Trypsin EDTA 2.5 g (solution A), 3-(4,5-Dimethylthiazol-2-Yl)-2,5-Diphenyltetrazolium-Bromide (MTT), Fluorescein Isothiocyanate Dextran 40 kDa (FD40), Dimethyl sulfoxide (DMSO), and HA powder (750–1000 kDa) were all purchased from Sigma Aldrich, Rehovot, Israel. Also, 24-well plates (Jet Biofil) and polyethylene (PET) tissue plate inserts (with a diameter of 6.5 mm, a surface area of 0.33 cm^2^, and a 0.4 μm pore size) (Jet Biofil) were purchased from Romical, Israel. Ethanol (by Carlo Alba) was purchased from Merck Life Science, Rehovot, Israel. Herbs of Kedem (Israel) supplied fresh ORD from Mount Hebron (1026 m above sea level). Dr. Josh Klein—from the Volcani Institute of the Israeli Agricultural Research Organization (Rehovot, Israel)—kindly provided the CM and CMC.

### 4.2. Extract Preparation

The extracts were prepared from the exterior epicarp of CM and CMC, while the leaves were used for ORD. First, the samples were washed in cold water, frozen at −80 °C, lyophilized for 48 h, and ground into powder. Next, 10 g of powder was dissolved in 100 mL of ethanol and mixed for 24 h. Then, the extracts were filtered through cellulose filter paper with 10–20 μm pores (by FiltraTECH, Saint-Jean-de-Braye, France). Lastly, the filtrate was concentrated under a vacuum at room temperature, fully lyophilized, and weighed to calculate the extract concentration.

### 4.3. Total Phenolic Content

The microplate total phenolic content (TPC) assay was adapted from the 96-well microplate fast blue BB method with minor modifications to measure the TPC in our extracts. To quantify the TPC, the absorbance of the control reaction (ethanol) was subtracted from the absorbance of the sample. Gallic acid solutions ranging from 50 to 1000 mg/L were standardized to create the calibration curve. First, 0.001 g of FBB was dissolved in 1 mL ethanol 100% (*v*/*v*), while 0.05 g of sodium hydroxide was dissolved in 1 mL DDW. Then, 10% of each solution (FBB and sodium hydroxide) was added to the test solutions, and 100 μL of each test solution (extracts or gallic acid as the reference solution) was placed in a flat-bottom 96-well microplate (NUNC, Roskilde, Denmark). This mixture was incubated for 90 min at room temperature. The absorbance was measured at 420 nm (using a Multiskan GO spectrophotometer from Thermo Fisher Scientific, Rehovot, Israel). Following the determination of the extract’s TPC in mg gallic acid equivalent (GAE) per mL via the spectroscopy analysis (Figure S1B), the TPC concentration was further normalized by the total initial weight of the extract used (Table S1). The TPCs for CM, CMC, and ORD were 0.2 ± 0.03, 0.2 ± 0.01, and 0.9 ± 0.03 GAE mg/ml and normalized to 2.8 ± 0.3%, 2.3 ± 0.15%, and 9.8 ± 0.4% GAE, respectively (see in Figure S1A).

### 4.4. TR146 Cell Culture Work

To evaluate the effect of the ORD, CM, and CMC extracts on the penetration of FD40 across the buccal epithelium, we used an in vitro model of TR146 cells grown on PET inserts. First, the standard human buccal epithelial cell line—TR146 (passages 8–11)—was cultivated in T75 flasks (Jet Biofil from Romical, Beer Sheva, Israel) at 37 °C, 5% CO_2_, and 95% humidity in a medium composed of high-glucose DMEM supplemented with 1% PS, 1% L-glutamine, and 10% FBS (termed complete DMEM medium (cDMEM). Then, the cells were detached using Trypsin–EDTA solution and seeded onto PET cell culture inserts (24-well plate) at 1.2·105 cells/cm^2^ per well. The cells were cultivated for 30 days, while the medium was replenished weekly. The upper compartment contained 0.3 mL, while the lower section contained 1 mL of complete medium.

### 4.5. Toxicity Assay

Since the extracts were prepared in ethanol, we first evaluated the potential toxicity of ethanol and the extracts on the viability of the TR146 cells. To this end, we used the MTT colorimetric viability assay [26,27]. First, 500 μL of complete medium containing 3 × 10^5^ cells/mL was seeded into each well (24-well plates) and incubated for 40 h. Afterward, the medium was replenished with 500 μL of the tested solution in each well. The tested solutions contained ethanol at different concentrations of 0%, 0.25%, 0.5%, 1%, 5%, 10%, and 20% *v*/*v* in a complete medium. The CM, CMC, and ORD extracts were tested at 0.1%, 0.3%, and 0.5% *w*/*v* in complete DMEM with 1% ethanol to maintain complete solubility. Each solution was tested in triplicate.

Next, the plates were incubated at 37 °C and 5% CO_2_ for an additional hour. Afterward, the media were removed, and the wells were washed with warm PBS and incubated for another 4 h with 450 μL of fresh complete medium and 50 μL of MTT reagent in each well. Cell viability was detected via a proliferation reagent, which is converted in live cells from yellow tetrazole MTT into purple formazan form by acellular reductase [8,28]. Then, 500 μL per well of DMSO was added to dissolve the purple crystals formed by the cells and incubated under the same conditions for another hour. Then, the 24-well plate with the dissolved formazan was measured at 550 nm using a plate reader (Agilent BioTek Synergy H1 Multimode Reader, Santa Clara, CA, USA). The cell viability was calculated using Equation (1) [4]:

(1) $$\mathrm{Cells}\;\mathrm{viability}=100\;[\frac{A_{t}{\;-\;A}_{b}}{A_{c}{\;-\;A}_{b}}]\;$$

where A_t_ is the absorbance of the cells after exposure to the tested solutions (ethanol or extracts), and A_b_ represents the blank solution’s absorbance, which consists of tested solutions like ethanol or extracts without cells. Meanwhile, A_c_ signifies the absorbance of the control, indicating cells being exposed solely to the complete medium (see Supplementary Information Figure S2).

### 4.6. In Vitro Buccal Permeability Measurements

As mentioned, the TR146 cells were grown for 30 days until the formation of full-confluency layers [8,18]. Then, the cells were exposed to the tested solutions for one hour at concentrations of 0.1%, 0.3%, and 0.5% *w*/*v* of CMC, CM, and ORD in a complete medium and 1% ethanol (*v*/*v*). After the exposure, the extracts were removed, and the cells were thoroughly washed twice with warm PBS. Then, the upper section was filled with 1 mg/mL of FD40 in complete medium without phenol red (Sigma, Rehovot, Israel). Samples of 0.1 mL were taken from the lower compartment at the following points: 0.25, 0.5, 1, 1.5, 2, 2.5, 3, 3.5, 4, 5, 6, 10, and 24 h post-exposure. The FD40 concentration in the samples was determined based on a calibration curve of FD40 in phenol-red-free complete medium and using a microplate reader (Agilent BioTek Synergy H1 Multimode Reader, San Jose, CA, USA) at 494 nm excitation and 521 nm emission wavelengths (y = 4 × 10^7^ × R^2^ = 0.9934; see Supplementary Information Figure S3).

The permeability coefficient is a key parameter in drug delivery, indicating the rate at which a drug crosses biological membranes [4,29]. This rate is influenced by factors such as the drug’s lipophilicity, molecular size, and the presence of specific transport mechanisms. Understanding permeability is crucial for predicting a drug’s absorption, distribution, metabolism, and excretion properties, all essential in drug development. The permeability coefficient is typically measured in units of velocity (cm/s or m/s), representing the speed at which the drug passes through a membrane [30,31]. PC provides a scale for the ease with which a specific substance permeates or passes through a particular tissue/membrane [32]. Many parameters can affect PC, such as temperature, pressure, molecular weight, hydrophilicity, membrane/tissue structures and compositions, the presence of PEs, etc. In this study, we evaluated the effect of our extracts on the PC of FD40 across the TR146 layer. The PCs were calculated using Equation (2) [33]:

(2) $$\;\mathrm{PC}\;[\frac{m}{s}]=\frac{{\;K}_{p}D}{s}=\frac{\frac{\mathrm{dQ}}{\mathrm{dt}}}{{A\;\times \;C}_{0}}$$

where D (m^2^/s) is the effective diffusion coefficient of a molecule (e.g., FD40) within the tested membrane (e.g., TR146 layers), K_p_ (dimensionless) is the partition coefficient between the medium and the tested membrane, h (m) is the thickness of the tested membrane, dQ/dt is the mass flow rate of the molecule across the tested membrane (kg/s), A is the cross-section of the insert (e.g., 3.36 × 10^−5^ m^2^), and C_0_ is the initial concentration of FD40 in the upper section. Effectively, PC can be calculated from the slope (of the linear range) of the normalized accumulated release (divided by C_0_ and A) vs. time (e.g., Figure 2).

### 4.7. Rapid Colorimetric Screening

A rapid colorimetric screening method was used to characterize the interaction of the tested extracts with the TR146 membrane. Rapid colorimetric screening can distinguish between surface interaction with membranal phospholipids, penetration into the bilayer (fluidizers), and damage to the lipid bilayer via extraction [4,19]. Briefly, 10,12-Tricosadiynoic acid (PDA monomer) was dissolved in chloroform and filtrated using a 0.45 μm PTFE membrane. Then, the chloroform was disposed of under a vacuum overnight. The white powder was dissolved with DMPC in ethanol/chloroform (1:1 vol) at a molar ratio of 3:2 (respectively). Next, the medium was evaporated under a vacuum for 48 h. The resulting powder was dispersed in double-distilled water to achieve a 1 mM total lipid concentration and sonicated (Q700 Sonica, Newtown, CT, USA) at 40% amplitude for 7 min (50% duty cycle). The solution was cooled and stored at 4 °C for at least 4 8 h before use. Finally, the solution was irradiated with UV light at 254 nm (Biolink-BLX, Vilber—Lourmat, Collégien, France) for 5 min in a Petri dish until the sample became intensely blue.

In a 96-well plate, 30 μL of lipid solution was mixed with 30 μL of the tested extract dissolved in 1% ethanol. Then, 30 μL of 25 mM Tris buffer (pH = 8) was added to each well to maintain a neutral environment. The baseline of the red color was determined using 1 M NaOH. The baseline of the blue color was determined using the vesicle/buffer solution without any supplement. The colorimetric response level was measured from the initial time point (0 min-PB_t=0_) at 5 min intervals for 20 min (PB_t=20_). The plate was read at 640 nm and 500 nm absorbance wavelengths using a plate reader (Synergy H1, Santa Clara, CA, USA). The colorimetric response (%CR) was calculated using Equations (3) and (4):

(3) $$\%\mathrm{CR}=\frac{{\mathrm{PB}}_{\mathrm{initial}\;}{-\;\mathrm{PB}}_{\mathrm{end}}}{{\mathrm{PB}}_{\mathrm{initial}}}\;\times \;100$$

(4) $${\mathrm{PB}}_{\mathrm{initial}\;\mathrm{or}\;\mathrm{end}}=\frac{A_{640\;\mathrm{nm}}}{A_{640\mathrm{nm}\;}{+A}_{500\;\mathrm{nm}}}$$

where A_i_ is the absorbance at the wavelength i.

As mentioned, this method indicates the nature of the tested materials with a phospholipid bilayer. No reaction between the phospholipids and the substance has occurred if no color change occurs. If a color change occurs immediately after exposure to the substance, this indicates a surface interaction. If the color changes take longer, the material penetrates the phospholipid bilayer. A loss of color suggests damage to the vesicles [19,29] (see Figure S5).

### 4.8. Statistical Analysis

Statistical analyses were performed using the statistics package GraphPad Prism 8 (GraphPad Software Inc., La Jolla, CA, USA), except for the principal component analysis (PCA), which was performed using JMP statistical discovery software (v.18.1.0, 2024). The Kruskal–Wallis test, followed by multiple comparisons corrected with Dunn’s method, was used to analyze the cell viability results after demonstrating an abnormal distribution according to the Shapiro–Wilk test. The permeability experiments were repeated at least four times. The constructed graphs present the accumulated mass of FD40 found in the receiver (i.e., bottom) compartment, divided by the insert cross-section and the initial concentration of FD40 in the upper compartment. The PC was obtained from the linear slope analysis for each tested solution.

A normal distribution was tested by using the Shapiro–Wilk test. The PC values were compared using the Brown–Forsythe ANOVA test (assumption of non-equal standard deviation), followed by multiple Dunnett comparisons for the PCs obtained from the linear range. The Kruskal–Wallis test, followed by various comparisons corrected with Dunn’s method, was used to analyze the colorimetric response results (*N* > 4); the Mann–Whitney test was applied between hyaluronic acid and HA+ ethanol. Statistical significance was denoted as * *p* < 0.05.

## Data Availability

The original contributions presented in the study are included in the article/Supplementary Material, further inquiries can be directed to the corresponding author.

## Supplementary Materials

The following supporting information can be downloaded at https://www.mdpi.com/article/10.3390/gels10120836/s1. Figure S1: Total Polyphenol Content of Ethanolic Extracts (Gallic Acid Equivalents), Figure S2: 24-well plates post one-hour exposure to MTT reagent. Figure S3: Calibration curve of FD40 in cDMEM. Figure S4: PC (after applying Mann-Whitney on DATA) between 1% *v*/*v* ethanol in cDMEM to cDMEM only post one-hour exposure. Figure S5: Normalized (see Equation (2)) accumulated FD40 in the receiving chamber that permeated through TR146 layers after one hour of exposure to the tested solutions and (HA, ethanol, HA with ethanol, and cDMEM. Figure S6: Normalized (see Equation (2)) accumulated FD40 for 24 h in the receiving chamber that permeated through TR146 layers after one hour of exposure to the tested extracts and control solutions in cDMEM. 0.1%, 0.3%, and 0.5% (*w*/*v*) of (S6A) CM in 1% ethanol in cDMEM, (S6B) CMC in 1% ethanol cDMEM, and (S6C) ORD in 1% ethanol and a cDMEM. And (6SD) control solutions (cDMEM, 1% *v*/*v* ethanol, 0.5% HA, 0.5% HA with 1% ethanol). Figure S7: Visual PDA vesicle’s -color- changes (in 96 wells plate) after exposure to different extracts and control solution (labels on the right) for 5 and 20 min; each well is labeled with the name of the extract/solution it was exposed to. Figure S8: Time dependence (for 20 min) of %CR. Values present the mean ± SD of *N > 4, and statistical* significances were denoted as ** p < 0.05* and *** p < 0.01* after applying Kruskal Wali’s test on all values, while Mann-Whitney test was used between hyaluronic acid and HA+ ethanol. Table S1: Mass concentration of the ethanolic extract stocks from 10 g of CM, CMC, and ORD. Table S2: The relative fluorescent intensity of different concentrations of FD40 at 494 nm excitation wavelength and 521 emission wavelength.
