# Supplementary material for: Natural Epithelial Barrier Integrity Enhancers—Citrus medica and Origanum dayi Extracts
Source: Gels. 2024 Dec 19;10(12):836. doi: 10.3390/gels10120836 (PMC11675818; doi:10.3390/gels10120836)
Supplement: Supplementary file 1 [file gels-10-00836-s001.zip › gels-3241900-supplementary.pdf]

Supplementary Information

1. Extracts Yields

Table S1 summarizes the mass concentration of the ethanolic extract stock solutions extracted from 10 g of material, while **Figure S1** presents the total phenolic content (TPC) of these extracts.

Table S1: Mass concentration of the ethanolic extract stocks from 10 g of CM, CMC, and ORD.

	Mass Concertation (mg/mL)
Citrus medica var Balady	90
Citrus medica var Calabria	60
Origanum dayi	50

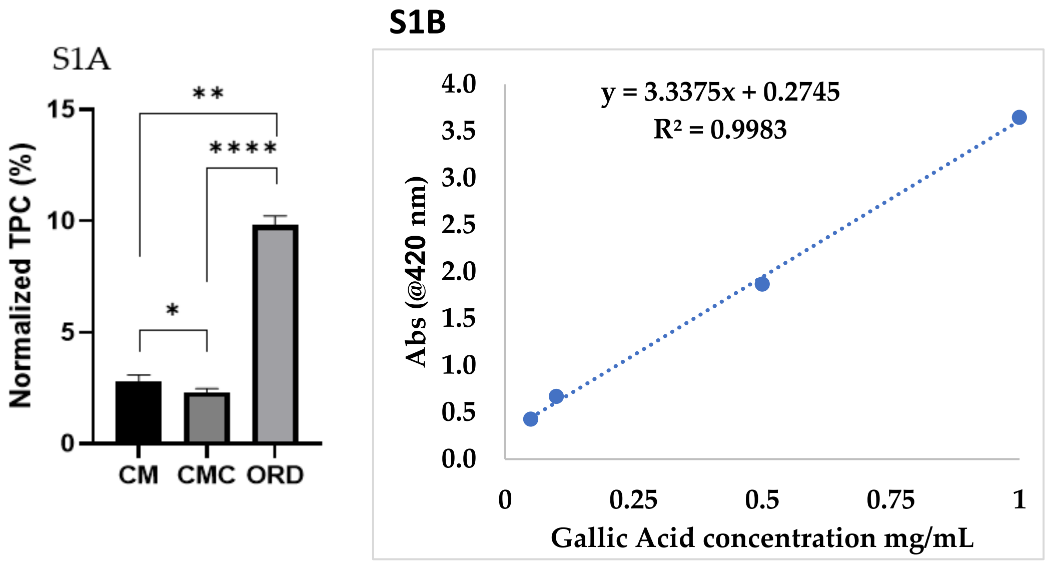

Figure S1: (**S1A**) Total Polyphenol Content of Ethanolic Extracts (Gallic Acid Equivalents), values presented are mean ± SD, *N>16* after applying the Kruska-Wallis test (nonparametric). significance was determined when **p<0.05, **p<0.01, ****p<0.001*. (**S1B**) calibration curve of gallic acid at 420 nm according to Fast Blue BB assay.

As can be seen from **Table S1** and **Figure S1**, TPC constitutes about 70% of ORD extract, while for CM and CMC, it is about 30%.

2. MTT Analysis

The MTT method was applied to test the potential toxicity of our extracts and ethanol. An example of the results can be seen in **Figure S2** below.

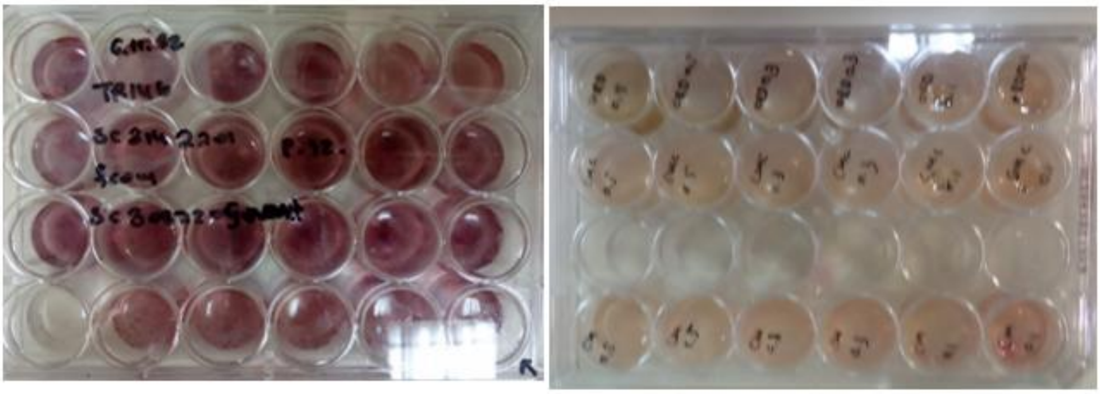

Figure S2: 24-well plates post-one-hour exposure to MTT reagent. The solution tested with cells is on the left, while the solutions tested without cells are on the right.

3. Calibration of FD40 on cDMEM

Calibration of FD40 in phenol red-free DMEM was required to assess the linear relation between the concentration and fluorescence of FD40

Table S2: The relative fluorescent intensity of different concentrations of FD40 at 494 nm excitation wavelength and 521 emission wavelength.

C mg/mL	Relative Fluorescent Units (RFU)
**0.25**	9014113
**0.125**	5249020
**0.0625**	2930110
**0.03125**	1612088
**0.015625**	840460
**0.0078125**	442304
**0.0039063**	239040
**0.0019531**	123191
**0.0009766**	63228
**0.0004883**	85541

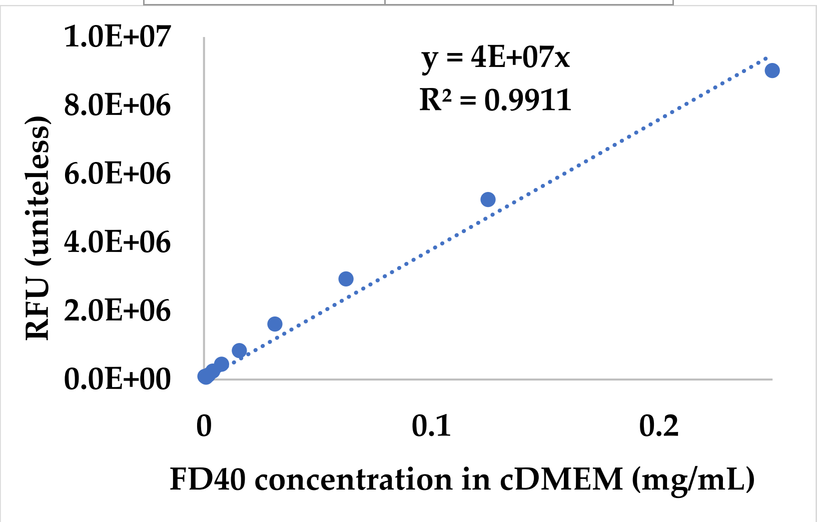

Figure S3: Calibration curve of FD40 in cDMEM.

4. *In vitro* Buccal Permeability Measurements

A comparison between the calculated PCs of 1% ethanol and complete DMEM was made to certify further that ethanol acts as a PE.

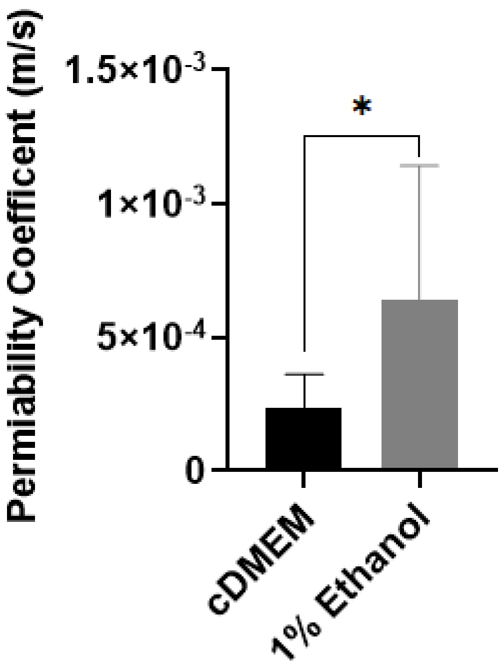

Figure S4: PC (after applying Mann-Whitney on DATA) between 1% v/v ethanol in cDMEM to cDMEM only post one-hour exposure. Mean ± SD, *N>4*, statistical significances were denoted as *$*p<0.05$* after applying the Brown Forsythe one-way ANOVA test**.**

It can be concluded from **Figure S4** that 1% ethanol can increase the PC of FD40 across TR146 *in vitro* after one h of exposure, effectively serving as a PE. Comparison of linear ranges of each control solution to HA 0.5% w/v with and without 1% ethanol.

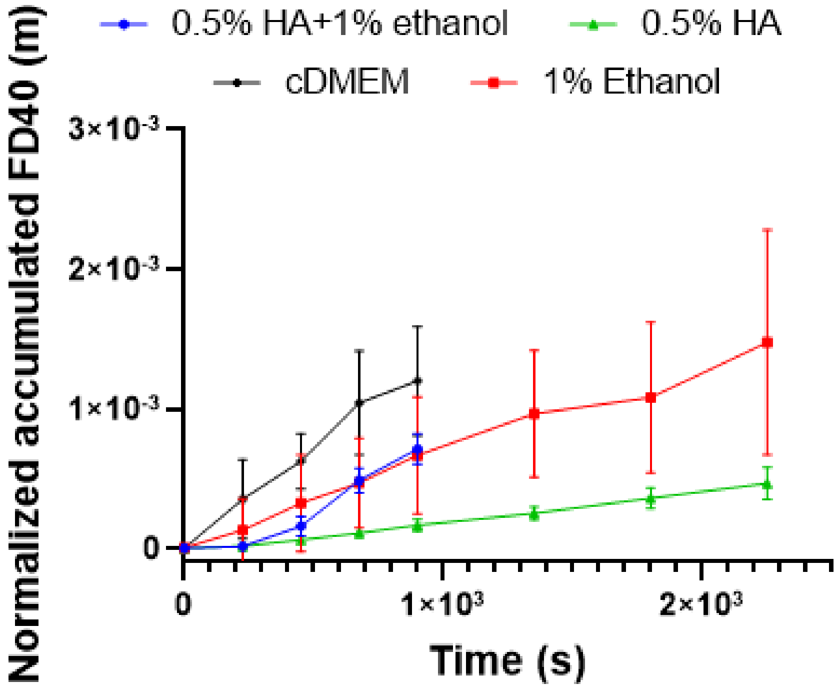

Figure S5: Normalized (see **Eq. 2**) accumulated FD40 in the receiving chamber that permeated through TR146 layers after one hour of exposure to the tested solutions and (HA, ethanol, HA with ethanol, and cDMEM. Values present the mean ± SD of *N*>4.

The linearity of each solution was maintained for 2 h, after which there was a significant decrease in FD40 concentration in the donner chamber, and the concentration gradient was not constant anymore (one of the assumptions leading to **Equation 2**). **Figure S5** shows the entire timeline for the different extracts and control solution concentrations. Note that PCs were obtained only from the linear regions where the assumptions of **Equation 2** are still valid.

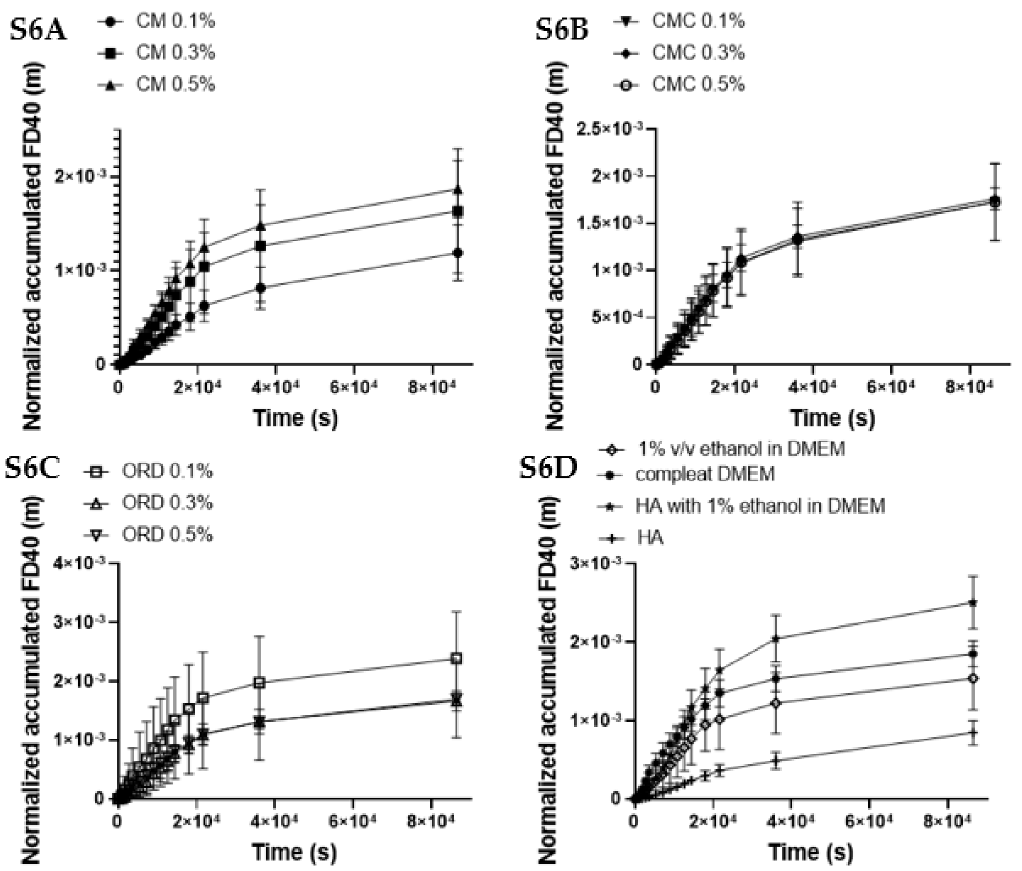

Figure S6: Normalized (see **Eq. 2**) accumulated FD40 for 24 h in the receiving chamber that permeated through TR146 layers after one hour of exposure to the tested extracts and control solutions in cDMEM. 0.1%, 0.3%, and 0.5% (w/v) of **(S6A)** CM in 1% ethanol in cDMEM, **(S6B)** CMC in 1% ethanol cDMEM, and **(S6C)** ORD in 1% ethanol and a cDMEM. And (**S6D**) control solutions (cDMEM, 1% v/v ethanol, 0.5% HA, 0.5% HA with 1% ethanol). Values present the mean ± SD of *N*>3.

5. Rapid Colorimetric Screening

The colorimetric response was calculated after measuring absorbance at 5 min of exposure to extracts and control solutions and after 20 min. In **Figure S7,** the color change of each solution is presented at 5 min and 20 min.

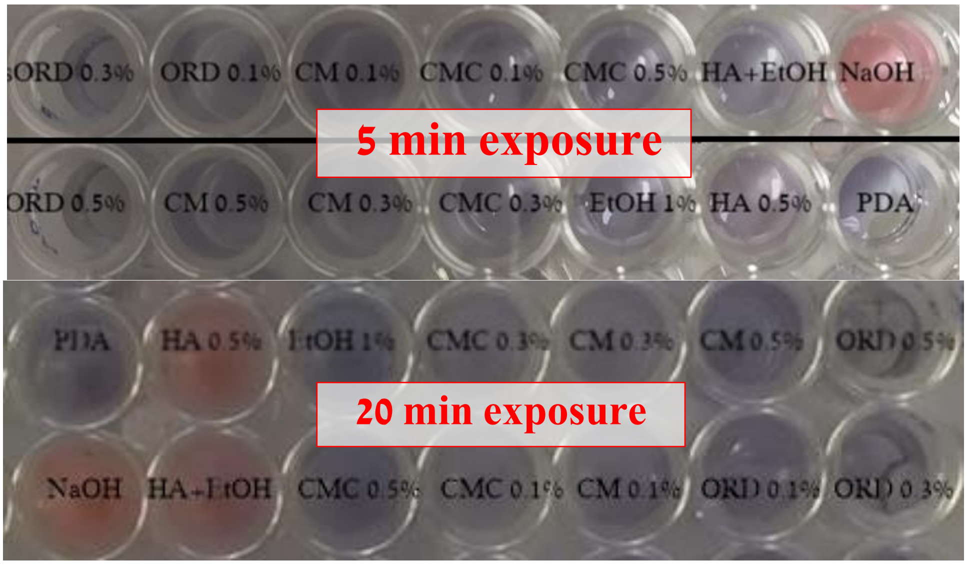

Figure S7: Visual PDA vesicle's -color- changes (in 96 wells plate) after exposure to different extracts and control solution (labels on the right) for 5 and 20 min; each well is labeled with the name of the extract/solution it was exposed to.

Colorimetric response compared to the initial point (t=0) depended on time duration for 1200 seconds. Only HA and HA+ ethanol show continuous increases in %CR from 300 to 1200 seconds.

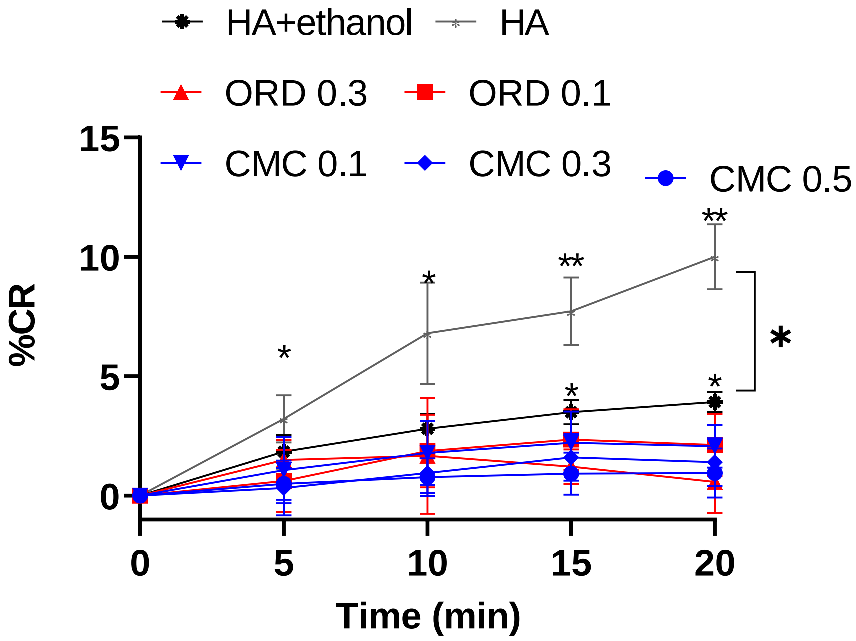

Figure S8: Time dependence (for 20 min) of %CR. Values present the mean ± SD of *N>4, and statistical* significances were denoted as $*p<0.05$ and $**p<0.01$ after applying Kruskal Wali's test on all values, while Mann-Whitney test was used between hyaluronic acid and HA+ ethanol.
